# Post-hospitalization remote monitoring for patients with heart failure or chronic obstructive pulmonary disease in an accountable care organization

**DOI:** 10.1186/s12913-023-10496-6

**Published:** 2024-01-13

**Authors:** Samantha Harris, Kayla Paynter, Megan Guinn, Julie Fox, Nathan Moore, Thomas M. Maddox, Patrick G. Lyons

**Affiliations:** 1 Washington University School of Medicine, St. Louis, MO USA; 2 BJC HealthCare, St. Louis, MO USA; 3 BJC Medical Group, St. Louis, MO USA; 4 Department of Medicine, Oregon Health & Science University, Portland, OR USA

**Keywords:** Remote patient monitoring, Innovation, Accountable care organization

## Abstract

**Background:**

Post-hospitalization remote patient monitoring (RPM) has potential to improve health outcomes for high-risk patients with chronic medical conditions. The purpose of this study is to determine the extent to which RPM for patients with congestive heart failure (CHF) and chronic obstructive pulmonary disease (COPD) is associated with reductions in post-hospitalization mortality, hospital readmission, and ED visits within an Accountable Care Organization (ACO).

**Methods:**

Nonrandomized prospective study of patients in an ACO offered enrollment in RPM upon hospital discharge between February 2021 and December 2021. RPM comprised of vital sign monitoring equipment (blood pressure monitor, scale, pulse oximeter), tablet device with symptom tracking software and educational material, and nurse-provided oversight and triage. Expected enrollment was for at least 30-days of monitoring, and outcomes were followed for 6 months following enrollment. The co-primary outcomes were (a) the composite of death, hospital admission, or emergency care visit within 180 days of eligibility, and (b) time to occurrence of this composite. Secondary outcomes were each component individually, the composite of death or hospital admission, and outpatient office visits. Adjusted analyses involved doubly robust estimation to address confounding by indication.

**Results:**

Of 361 patients offered remote monitoring (251 with CHF and 110 with COPD), 140 elected to enroll (106 with CHF and 34 with COPD). The median duration of RPM-enrollment was 54 days (IQR 34–85). Neither the 6-month frequency of the co-primary composite outcome (59% vs 66%, FDR *p*-value = 0.47) nor the time to this composite (median 29 vs 38 days, FDR *p*-value = 0.60) differed between the groups, but 6-month mortality was lower in the RPM group (6.4% vs 17%, FDR p-value = 0.02). After adjustment for confounders, RPM enrollment was associated with nonsignificantly decreased odds for the composite outcome (adjusted OR [aOR] 0.68, 99% CI 0.25–1.34, FDR *p*-value 0.30) and lower 6-month mortality (aOR 0.41, 99% CI 0.00–0.86, FDR p-value 0.20).

**Conclusions:**

RPM enrollment may be associated with improved health outcomes, including 6-month mortality, for selected patient populations.

**Supplementary Information:**

The online version contains supplementary material available at 10.1186/s12913-023-10496-6.

## Background

Patients with chronic medical conditions such as congestive heart failure (CHF) or chronic obstructive pulmonary disease (COPD) face disease-related mortality and morbidity including hospitalization and decreased quality of life [1–4].Approximately 1 million heart failure hospitalizations occur annually in the US, with a 20% readmission rate within 30 days [5, 6]. Barriers to effective care including limited access, symptom recognition, understanding, and medication adherence [7].

Remote patient monitoring (RPM) offers the potential to improve care and outcomes for patients with chronic conditions by addressing these challenges. RPM is a technology-enabled healthcare delivery model which allows providers to gather data and manage patients’ health outside of traditional healthcare settings. Mechanistically, RPM may improve access, promote patient self-management, detect early warning signs of clinical decompensation, and facilitates timely preventative or rescue interventions before hospitalization [8].

Prior studies of RPM in patients with chronic conditions have yielded mixed findings regarding hospital admission and mortality [9–13], potentially due to differences in the chronic condition studied, RPM intervention (e.g., monitoring frequency), and outcomes measured. Further, the extent to which RPM interventions (intended to focus on high-risk patients with chronic conditions) might benefit patients enrolled in accountable care organizations (ACOs), which have been shown to reduce hospitalizations regardless of patient risk, remains unclear [14]. In the US, ACOs are groups of clinicians and facilities jointly providing care for a defined group of patients (e.g., geographically, or with a specific condition) with the intent to reduce fragmentation improve care coordination across a health system, improve quality, and improve outcomes [15, 16]. ACOs are financially incentivized by Medicare to improve quality and outcomes and reduce spending, sharing in a proportion of cost savings and paying penalties if they provide fragmented, more costly care. We implemented and evaluated a post-hospitalization RPM program for patients with chronic conditions within a large academic hospital system’s ACO. We hypothesized that RPM participation would decrease all-cause readmission, mortality, and emergency department visits in this high-risk patient population.

## Methods

### Design, setting, and study population

This was a nonrandomized prospective study of adult patients enrolled in BJC HealthCare’s ACO. The ACO serves over 70,000 patients Medicare and Medicare Advantage patients in the St. Louis, MO, metropolitan area. All hospitalized ACO-enrolled patients enter a care transition program for 30 days upon hospital discharge, which includes medication review, disease-specific education, scheduling follow-up appointments, and addressing barriers to care (e.g., assigning chore workers). The ACO established an RPM program in February 2021, offering RPM to eligible patients on hospital discharge in addition to and concurrent with existing care transition services for those who elected to enroll.

RPM eligibility required medically-diagnosed CHF, COPD, or a recommendation from the patient’s primary care provider for another chronic condition. Exclusion criteria for RPM eligibility were (1) discharge to hospice, (2) outpatient dialysis, and (3) a screening nurse’s determination that comorbid or socioeconomic obstacles (e.g., moderate to severe cognitive impairment, unstable housing) would preclude participation. Patients who elected not to enroll in RPM served as controls post hoc.

Because over 96% of eligible patients had diagnoses of CHF or COPD, and because we expected the potential benefit of RPM to vary by condition, we restricted our evaluation to RPM-eligible patients with CHF or COPD. The study focused on RPM-eligible patients who were offered enrollment between February 2021 and December 2021, with a planned 30-day minimum for RPM and a predetermined 6-month follow-up period (because we expected that some potential benefits of increased monitoring might accrue past the actively-monitored period).

This RPM program was initiated as an ACO-sponsored quality improvement activity. The post hoc study described here was designed subsequent to RPM program development and implementation. The Washington University Institutional Review Board reviewed and approved the analysis protocol (#202207118) prior to data collection and analysis. All methods were performed in accordance with relevant guidelines and regulations.

### RPM intervention

The ACO provided RPM personnel and services using materials and equipment from a commercial vendor (Health Recovery Systems, Hoboken, NJ). The vendor managed distribution and between-patient cleaning of devices and materials but played no role in the design, oversight, or analysis of this study.

ACO staff contacted eligible patients within 48 hours of discharge to offer RPM enrollment. To preclude immortal time bias, the 6-month follow-up period began on the day of ACO staff contact for all patients. Enrolled patients were mailed an RPM kit containing a Samsung tablet preloaded with RPM software and cellular capabilities (to allow patients without home internet to participate), an A&D blood pressure monitor and cuff, scale, pulse oximeter, and written set-up instructions. All items were Bluetooth-enabled to sync with the tablet. Tablets included preloaded educational video about the relevant chronic condition.

During participation, tablet devices alerted once daily to prompt participants to record vital signs and complete surveys. Abnormal vital signs or affirmative survey responses (Supplementary Table 1) triggered real-time alerts to assigned ACO nurses who then called the patient for further triage.

ACO nurses assessed the need for continued enrollment after 30 days; patients who had not had an RPM alert within the prior 7 days were eligible, but not required, to “graduate” from the program (i.e., each nurse had discretion to recommend continued enrollment based on their clinical judgment and the patient’s desires). This allowed the limited number of RPM kits to be redistributed. Patients could disenroll from the program at any time. Hospital admission paused RPM enrollment, which resumed automatically at discharge.

### Data acquisition and management

Demographics, insurance, comorbidities, social history, medications, procedures, and encounter data were extracted from the electronic health record (EHR; Epic, Verona, WI) through direct chart review. Because the other major health systems in the ACO’s catchment area use the same EHR, data from other health systems were extracted could also be we had access to the timing and details of essentially all local healthcare encounters via the vendor’s “Care Everywhere” interoperability feature (i.e., it is unlikely that patients experienced an outcome that we could not identify from their chart). Daily vital signs and surveys metadata were extracted from the RPM vendor for an exploratory *post-hoc* analysis.

### Outcomes

We prespecified two primary outcomes for this analysis: (1) the composite of death, hospital admission, or emergency care not resulting in an admission within 180 days of RPM eligibility, and (2) the time to occurrence of this composite. Secondary outcomes included individual component of the composite, death or hospital admission, time to death or hospital admission, number of specialist office visits, number of nonspecialist office visits, and length of hospital stay for admitted patients. The 6-month follow-up period started on the day of RPM eligibility.

### Covariates

Clinical data was measured at the time of RPM eligibility (e.g., subsequently-assigned comorbid diagnoses were not recorded). Because of uncertainty regarding how frequently social determinants of health (e.g., food insecurity) are recorded in discrete EHR fields [17], we considered each patient to have any individual insecurity if they had been recorded as having such.

We recorded the presence or absence of prescriptions in the following classes: (1) beta-blocker, (2) angiotensin-converting enzyme inhibitor, angiotensin II receptor antagonist, or angiotensin receptor neprilysin inhibitor, (3) mineralocorticoid receptor antagonist, (4) sodium/glucose cotransporter-2 inhibitor, (5) inhaler, and (6) insulin.

### Analyses

Continuous data were presented as median (IQR), and categorical data as n (percent). Unadjusted comparisons between RPM enrollees and RPM-eligible control patients were made using Wilcoxon rank sum tests, Pearson’s Chi-squared tests, and Fisher’s exact test as appropriate. We produced Kaplan-Meier curves to visualize time to the primary outcome for each group and compared these using the log-rank test.

Because patients self-determined their enrollment in RPM in this nonrandomized study, we expected significant confounding by indication in terms of the relationship between RPM participation and patient outcomes [18]. Hence, we elected *ex ante* to compare categorical outcomes between RPM participants and control patients through doubly robust estimation [19]. This approach combines propensity score estimation (i.e., the conditional likelihood that a patient would be in the exposure group, based on observed characteristics) with traditional multivariable logistic regression such that the final effect estimator is robust to misspecification of either model.

A priori, we used a directed acyclic graph to prespecify, through study team consensus, relevant potential confounders (i.e., variables likely to be associated with both the decision to enroll in RPM and relevant outcomes, but not on the causal pathway between them) [20]. These confounders were primary diagnosis (CHF vs COPD), age (modeled as a continuous linear variable), gender, insecurities related to housing, food, or living expenses (modeled as having any insecurity vs having no insecurities), current or prior use of tobacco products (modeled as ever vs never), number of healthcare encounters (admissions, ED visits, or office visits, to approximate baseline healthcare utilization) in the prior year to study eligibility, prescriptions of the previously listed medication classes, and individual comorbidities (Supplementary Table 2).

Analysis of time-to-event data in the setting of confounding by indication is an emerging methodological area [21–23]. Under the assumption that such confounding would be present at baseline and not time-varying over the course of the study, we used the same potential confounders in a multivariable logistic regression model to obtain propensity scores (i.e., each patient’s model-derived propensity to choose RPM enrollment). We then fit Cox proportional-hazards models to estimate the adjusted relationship between RPM enrollment and time to the primary outcome with these propensity scores included as a covariate. Although this process does not directly employ a doubly robust estimator, it may be more accurate than other common propensity-based approaches (e.g., inverse probability of treatment weighting) if confounding by indication is strong [24].

### Subgroup and sensitivity analyses

To contextualize and strengthen our findings, and because patients with CHF and COPD may have differential mechanisms of potential benefit from RPM [25], we repeated our analyses in each of these cohorts separately.

Next, because social determinants of health are (a) likely to confound the observed relationship between RPM enrollment and clinical outcomes and (b) frequently missing with unclear missingness patterns, we prespecified several sensitivity analyses to test the robustness of our findings to these challenges. First, we imputed all missing data regarding insecurities in housing, food, or living expenses for our baseline case as “no.” Second, we set all missing data to “yes,” and last, we set missing data to be “yes” or “no” contingent on the presence or absence of the primary outcome.

In an exploratory post hoc sensitivity analysis, we performed the observational analog of a per-protocol analysis [26, 27], in which we considered the degree of RPM engagement as a component of the exposure. We performed multivariable logistic regression treating RPM exposure as a continuous variable between 0 and 1, based on proportion of completed surveys and vital sign recordings in vendor data. We then performed doubly robust estimation on a cohort in which RPM exposure was redefined by adherence of 90% or greater to any single RPM metric.

We performed all analyses using R 4.2.1 (R Project for Statistical Computing, Vienna, Austria) and the tidyverse, drgee, survival, and survminer packages [28–31]. We adjusted *p*-values by the false discovery rate (FDR) methodology suggested by Benjamini & Hochberg [32], and considered FDR *p*-values < 0.05 statistically significant.

## Results

### Patient characteristics

Between February 2021 and December 2021, 375 patients were offered ACO RPM enrollment; of these, 212 had CHF and 150 had COPD (Supplementary Fig. 1). We excluded 14 patients due to uncommon enrollment diagnoses. Compared to eligible patients who did not enroll in RPM (*n* = 221 [145 CHF, 76 COPD]), patients who enrolled (*n* = 140 [106 CHF, 34 COPD]) were younger (median age 74 [IQR 66–83] vs 76 [70–84], *p* = 0.02), more likely to be nonsmokers (31% vs 19%, *p* = 0.01), and more likely to have Medicaid (22% vs 4.1%, *p* < 0.01) (Table 1). Baseline comorbidities, medications, and healthcare utilization were similar between groups. Median participation duration for enrolled patients was 54 [IQR 34–85] days (Table 2).

**Table 1:** Patient characteristics

	Control, *N* = 221	RPM, *N* = 140	*p*-value
***Baseline Demographics***			
Age, median (IQR)	76 (70, 84)	74 (66, 83)	0.02
Female gender, n (%)	114 (52)	86 (61)	0.07
Race, White, n (%)	173 (78)	99 (71)	0.10
Race, Black, n (%)	48 (22)	41 (29)	0.10
Any Insecurity, n (%)	26 (12)	18 (13)	0.80
Smoking, ever, n (%)	180 (81)	96 (69)	0.01
RPM eligibility for CHF, n (%)	145 (66)	106 (76)	0.04
Medicaid, n (%)	9 (4.1)	31 (22)	< 0.001
***Baseline Health Care Utilization Year Prior to Admission***, median (IQR)			
Number of Admissions	2 (1, 3)	2 (1, 3)	0.50
Number of Office Visits	7 (4, 12)	9 (6, 12)	0.04
Number of Specialist Visits	3 (1, 7)	3 (1, 7)	0.40
Number of ED Visits without Admission	0 (0, 1)	0 (0, 1)	0.40
***Baseline Medical Diagnoses, n (%)***			
Systolic Heart Failure	81 (37)	60 (43)	0.20
Diastolic Heart Failure	129 (58)	85 (61)	0.70
COPD	126 (57)	80 (57)	> 0.90
Atrial Fibrillation	105 (48)	57 (41)	0.20
Hypertension	202 (91)	126 (90)	0.70
Coronary or Peripheral Arterial Disease	150 (68)	72 (51)	0.002
Diabetes	89 (40)	73 (52)	0.03
Obesity	98 (44)	70 (50)	0.30
Chronic Kidney Disease	146 (66)	94 (67)	0.80
Cancers, excluding non-metastatic skin cancers	55 (25)	32 (23)	0.70
***Medications at Time of Cohort Entry,*** median (IQR)			
Total Number of Prescriptions	14 (11, 18)	15 (12, 19)	0.14
Total GDMT Meds	1 (1, 2)	2 (1, 2)	0.02
Inhaler	1 (0, 2)	1 (0, 2)	0.20
Insulin	0 (0, 0)	0 (0, 0)	0.14

Baseline demographics, medical diagnoses, and medications extracted at the time of cohort eligibility. Health care utilization measured between 1year prior to cohort eligibility and day of cohort eligibility. Pearson’s Chi-squared test and Wilcoxon Rank Sum Test used for categorical and continuous data, respectively

**Table 2:** Adherence to RPM

	Adherence, Median (IQR)	Readings Taken, Median (IQR)
Blood pressure	86% (60, 97)	43 (25, 65)
Pulse	86% (62, 97)	42 (24, 63)
Weight	88% (58, 97)	42 (23, 67)
Survey	68% (37, 92)	35 (16, 56)
RPM Duration, days	54 (34, 85)	

Adherence data to each RPM metric for enrolled patients. Adherence measured as daily vital sign logging and survey submission. Of note, if a patient was admitted to the hospital during their RPM enrolled time, this time was excluded from adherence calculations. Reported as median and IQR for % adherence, and number of readings taken or days’ duration

### Unadjusted outcomes

**Fig. 1:**
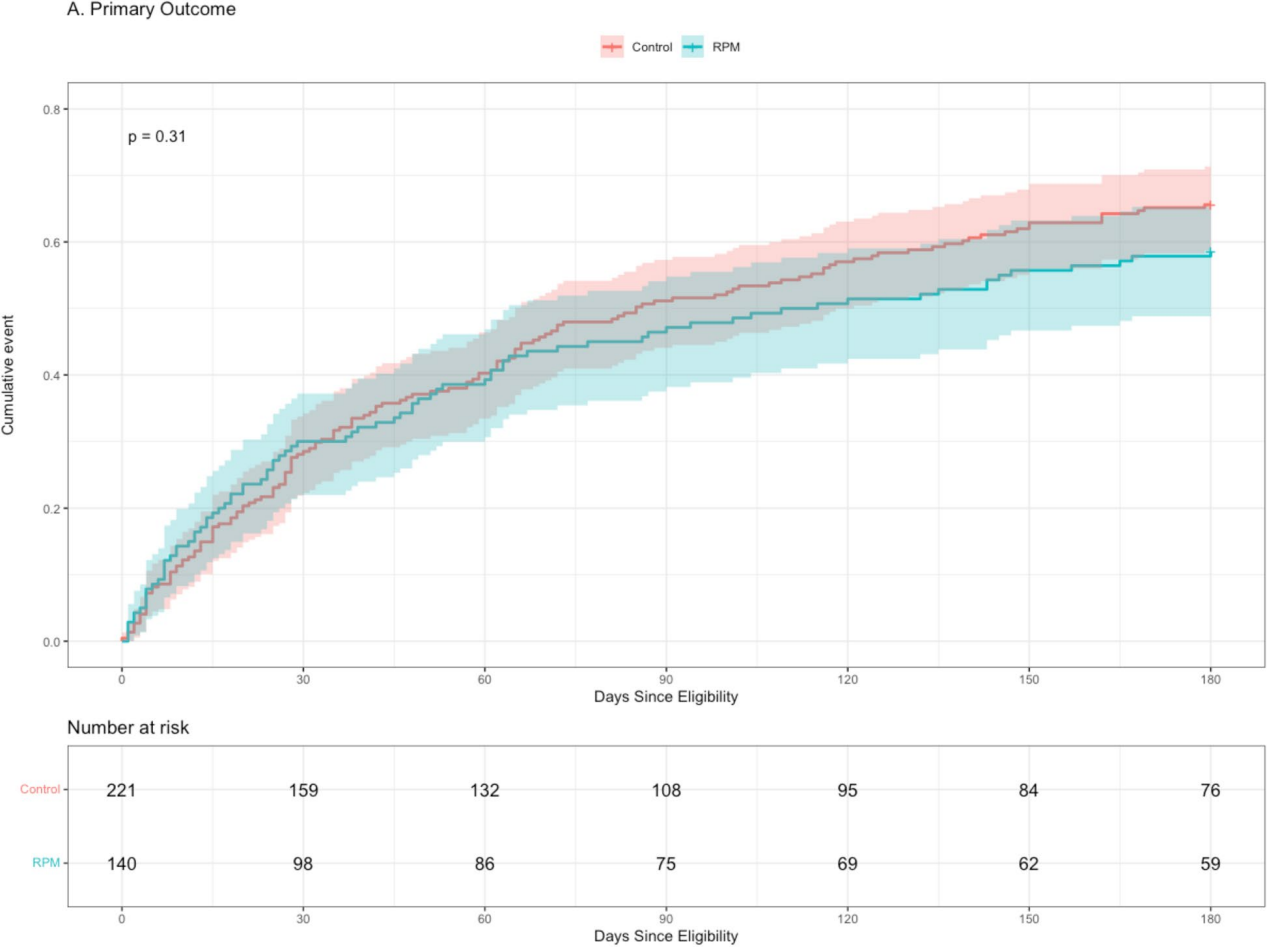
Cumulative incidence plot of time to death, hospital admission, or emergency department visit. RPM, remote patient monitoring. RPM: Remote patient monitoring

Neither the 6-month frequency of the co-primary composite outcome (Table 3) nor the time to this composite (Fig. 1) differed between the RPM and control groups. However, the RPM group had lower 6-month mortality (6.4% vs 17%, FDR *p*-value = 0.02). The RPM group had more overall (median 6 [IQR 4–8] vs 4 [2–7], *p* = 0.02) and subspecialty (median 6 [IQR 4–8] vs 4 [2–7], FDR *p* value = 0.02) outpatient encounters during the follow-up period. The RPM group had a trend towards fewer time in days to first ED visit (median 42 [IQR 20–94] vs 73 [31–112], FDR *p*-value = 0.18).

**Table 3:** Unadjusted outcomes

	Control, *N* = 221	RPM, *N* = 140	FDR *p*-value
Composite Outcome	145 (66)	82 (59)	0.47
Admission or Death	124 (56)	68 (49)	0.47
Death	37 (17)	9 (6.4)	0.02
ED Visit	59 (27)	41 (29)	0.75
Admission	113 (51)	67 (48)	0.70
Total Length of Stay for Admissions, days	6 (3, 14)	8 (4, 16)	0.62
Num. Office Visits in 1 Month	1 (1, 2)	1 (1, 2)	0.62
Num. Office Visits in 6 Month	4 (2, 7)	6 (4, 8)	0.02
Num. Office Visits with Specialist in 6 months	4 (2, 7)	6 (4, 8)	0.02
Time to Composite Outcome, days	38 (15, 83)	29 (11, 71)	0.60
Time to Admission or Death, days	39 (15, 82)	41 (12, 86)	0.75
Time to Death, days	72 (42, 124)	88 (37, 98)	0.75
Time to ED Visit, days	73 (31, 112)	42 (20, 94)	0.18
Time to Admission, days	38 (15, 84)	43 (13, 87)	0.80

Outcomes were measured for 6 months following time of RPM eligibility. The primary outcomes are a composite of (1) hospital admission, (2) death, (3) ED visit not resulting in hospital admission, and time to composite outcome. Presence or absence of outcome specified as n, (%). All other data reported as median (IQR). Pearson’s Chi-squared test and Wilcoxon Rank Sum Test used for categorical and continuous data, respectively

### Adjusted analyses

**Fig. 2:**
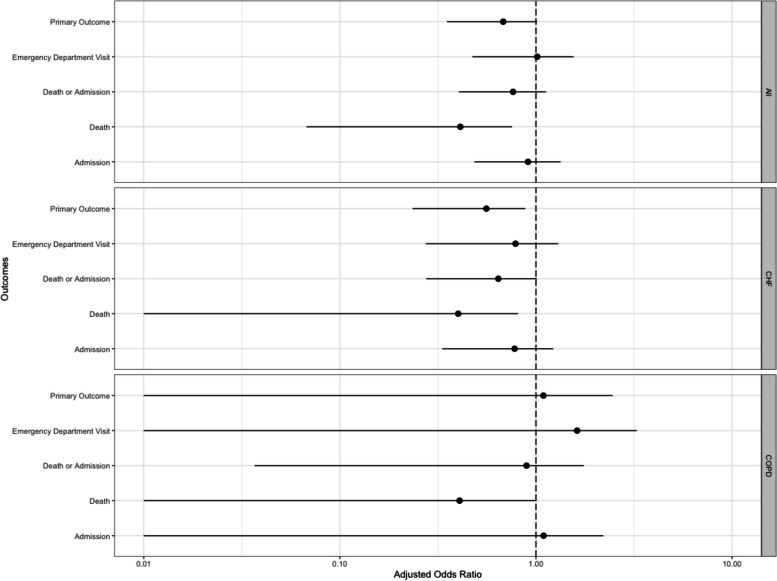
Adjusted primary and secondary outcomes (via doubly robust estimation) for overall population and sub-populations. CHF, congestive heart failure; COPD, chronic obstructive pulmonary disease

After doubly-robust estimation to adjust for prespecified confounders, including confounding by indication, we found nonsignificantly decreased odds for the primary composite outcome (adjusted odds ratio [aOR] 0.68, 99% CI 0.25 to 1.11, FDR *p*-value = 0.30), and a decrease in the 6-month mortality (aOR 0.41, 99% CI 0.00 to 0.86, FDR *p*-value = 0.20) that did not reach FDR-adjusted statistical significance. The propensity-adjusted time-to-event analysis showed no significant risk for the composite outcome in the study cohort compared to the control group (adjusted HR [aHR] 1.07, 99% CI 0.74 to 1.44, FDR *p*-value = 0.90), but did show a non-significant decrease in time to first ED visit (aHR 1.79, 99% CI 0.99 to 3.26, FDR *p*-value = 0.05). Adjusted analysis for secondary outcomes generally followed this pattern (Fig. 2**,** Table 4).

**Table 4:** Adjusted outcomes

*Doubly Robust Regression Analysis*				
	**aOR**	**99% Low CI**	**99% High CI**	FDR *p*-value
Composite of Death, Admission, or ED Visit	0.68	0.25	1.11	0.30
Admission or Death	0.76	0.29	1.24	0.43
Death	0.41	0.00	0.86	0.20
ED Visit	1.01	0.30	1.73	0.96
Admission	0.91	0.35	1.47	0.86
*Time to Event Analysis*				
	**HR**	**99% Low CI**	**99% High CI**	FDR *p*-value
Composite of Death, Admission, or ED Visit	1.07	0.74	1.55	0.90
Admission or Death	1.02	0.68	1.54	0.90
Death	2.20	0.44	11.10	0.53
ED Visit	1.79	0.99	3.26	0.05
Admission	1.02	0.66	1.58	0.90

Doubly robust estimation was used for binary outcomes and Cox proportional hazard models with adjustment for logistic-estimated propensity scores were used for time-to-event outcomes

### Subgroup analysis

In the subgroups of RPM-eligible patients with CHF (*n* = 251 [106 RPM, 145 control]) and COPD (*n* = 110 [34 RPM, 76 control]), patient characteristics (Supplementary Table 3) and outcomes (Supplementary Tables 4 and 5) were similar to those of the overall cohort. In the CHF group, the unadjusted RPM-associated 180-day mortality was lower (6.6% vs 17%, FDR *p*-value = 0.05). The adjusted primary outcome and 6-month mortality in the CHF group were both decreased.

### Sensitivity analyses

Sensitivity analyses in which we quantified social determinants of health through different approaches yielded similar results to the primary analysis (Supplementary Table 6). When the missing data is set based on the outcome, the adjusted OR for disk loses significance.

Among RPM-enrolled patients, those without the primary outcome had higher adherence to logging vital signs and symptoms in the RPM portal (BP Adherence 94% vs 74%, *p* < 0.01) (Supplementary Table 7). Duration of enrollment did not differ between these groups (*p* = 0.70). In an exploratory analysis based on these findings, RPM engagement was associated with decreased adjusted odds for the composite outcome (aOR 0.34, 99% CI 0.04–0.65). When defining adherence as greater than 90% for any measurement, we found that RPM enrollment was associated with decreased odds for all outcomes except ED visit (Supplementary Table 8).

## Discussion

This prospective analysis of ACO patients with CHF or COPD found that RPM enrollment was associated with significantly lower unadjusted mortality, decreased adjusted odds of 6-month mortality, and nonsignificantly decreased adjusted odds for the primary composite outcome of death, rehospitalization, or emergency care. These findings suggest there may be benefit of RPM interventions for selected populations. Critically, there was no significant increase for time-to-event in our cohort, an expected finding given that the benefit of RPM should not be instantaneous.

These results, primarily driven by patients with CHF, align with prior work demonstrating a positive relationship between RPM and decreased hospitalizations and mortality [11, 13, 33–36]. However, they contrast with several randomized trials which did not demonstrate clear benefits from RPM [9, 37, 38]. Such a contrast might be considered unsurprising, given the complexity and heterogeneity in most chronic conditions. Some flares of chronic conditions, for example shortness of breath in heart failure or uncontrolled sugars in diabetes, may allow for actionable responses through early detection, thus offering greater potential for improvement with RPM. The mixed benefit seen in prior studies may also be due to the heterogeneity in RPM interventions: there are many non-standardized variables that have been implemented across studies including the types of data collected, data collection methods, and the frequency and timeliness of data transmission to health care providers for actionable responses.

Conflicting RPM benefit may also be due to the complexity of outcome selection. While we selected a composite primary outcome to maximize statistical power and our follow-up period extended past the RPM duration period to capture lagging indicators, it seems likely that enrollment in RPM had an impact on how patients interacted with health care. For example, RPM-enrolled patients had a decreased time to first ED visit, while risk for any ED visit was unchanged. This may suggest that RPM alerts facilitate early detection and timely intervention, enabling patients to receive appropriate care sooner. Additionally, RPM-enrolled patients had increased outpatient office visits which was not associated with lower risk for hospitalizations or ED visits, but may have contributed to the observed mortality reduction. Thus, selecting outcomes and measurement frequency that more accurately capture changes in disease state may inform the findings of future RPM studies.

Our findings were robust to multiple sensitivity analyses, including an exploratory investigation into the possibility of “dose-dependence” in terms of RPM’s potential benefit. Interestingly, patients who experienced the composite outcome also had lower RPM usage, while those who did not experience an outcome more frequently recorded their symptoms and vital signs. In adjusted analysis, the RPM “dose” was positively associated with the adjusted odds of the primary outcome, suggesting that increased usage improves overall management of patients’ chronic conditions. Indeed, a prominent recent negative study of RPM in heart failure described overall lower engagement (~ 80%) than the rates in our study [38]. Future research should strive to explicate the mechanisms by which RPM works in terms of improving outcomes, determine the extent to which implementation strategies and duration of follow-up contribute to particular benefits.

The largest limitation to this work is its nonrandomized nature and the likelihood of confounding by indication. Notably, baseline comorbidities, medications, and healthcare utilization were similar between the RPM and control groups, suggesting that such confounding may not have been as strong as anticipated. Further, modern methods, including doubly robust estimation, offer an opportunity to minimize the bias from such confounding. However, even these methods cannot account for unmeasured confounders. Ultimately, confounding by indication will depend on the extent to which unmeasured factors (e.g., trust in the health care system) contribute causally to outcome differences based on RPM enrollment. An additional limitation is that mortality and health care utilization incompletely reflect of a patient’s overall health; without important outcomes such as patient-reported measures and costs, we risk misestimating this program’s impact. Third, we did not systematically collect patients’ reasons for RPM discontinuation (i.e., before 30 days). Because these reasons might be closely tied to patient outcomes (e.g., discontinued because of low motivation, discontinued because of entry into a long-term care facility), they represent an important variable to collect in future projects, as well as an important barrier to adoption. Just as early discontinuation may have limited our findings, so too could have our choice to offer patients “graduation” after 30 days. While our intent in this decision was to maximize the total number of patients offered RPM, doing so could have biased our results towards the null.

This study has several notable strengths. First, doubly robust regression is a modern and sophisticated approach to account for confounding by indication, increasing our confidence in these findings. Second, our strict control for potential false discovery helps minimize over-interpretation of our findings. Third, we achieved complete data capture via detailed chart review and vendor data extraction; because of our specific study population (and the availability of outcomes from other health systems via our EHR), we likely achieved complete capture of outcomes as well. Fourth, our deliberate and pragmatic approach to RPM enrollment (e.g., accounting for lack of home internet) allowed us to include a diverse group of patients.

In conclusion, RPM enrollment was associated with decreased adjusted odds of 6-month mortality in this prospective observational study of post-hospitalization patients with CHF and COPD. These findings suggest RPM interventions may have benefit for selected populations.

### Supplementary Information

**Additional file 1.**

## Data Availability

While raw data containing protected health information are unable to be shared, the final analysis dataset will be available at [link to come] on date of publication. The datasets generated and analysed during the current study are not publicly available because they contain protected health information and patient identifiers, but are available from the corresponding author on reasonable request.
