# Supplementary material for: Post-hospitalization remote monitoring for patients with heart failure or chronic obstructive pulmonary disease in an accountable care organization
Source: BMC Health Serv Res. 2024 Jan 13;24:69. doi: 10.1186/s12913-023-10496-6 (PMC10787416; doi:10.1186/s12913-023-10496-6)
Supplement: Supplementary file 1 — Additional file 1. [file 12913_2023_10496_MOESM1_ESM.docx]

Supplementary Figure 1. Cohort inclusion diagram.


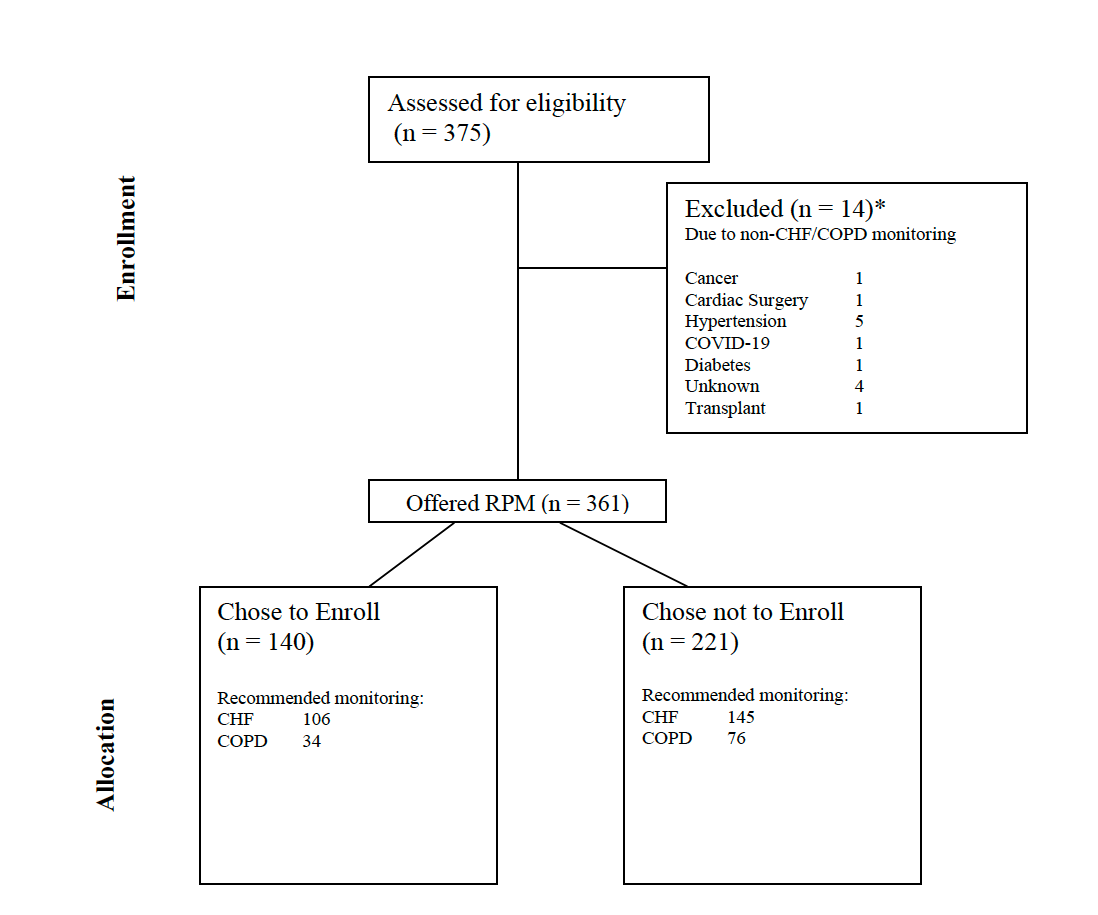


**Supplementary Table 1.** Vital sign parameters and survey questions. If a vital sign is recorded that is outside the defined parameters, or if no vital signs are recorded, then an alert is triggered. If any survey question is answered yes, an alert is triggered.

| ***Vital Sign Triggers*** | ***Survey Triggers*** |
| --- | --- |
| More than 3 lbs of weight gain in 24 hours | Have you experienced any increased Shortness of Breath with daily activity in the past 24 hours |
| More than 5 lbs of weight gain in 7 days | Have you had any increased or new swelling in your ankles, feet, or abdomen in the last 24 hours? |
| A change of 10 lbs of weight from baseline (gain or loss) | Have you had any increased or new coughing or wheezing in the last 24 hours? |
| No weight reported for 1 consecutive day | Have you experienced any increased tiredness or fatigue in the last 24 hours? |
| Systolic blood pressure measurement below 90 or above 160 | Have you had any increased or new nausea or lack of appetite in the last 24 hours? |
| Diastolic blood pressure measurement below 40 or above 90 | Have you had any problems thinking clearly in the past 24 hours? |
| No blood pressure measurement reported for 1 consecutive day | Have you felt your heart racing faster than normal in the past 24 hours? |
| Heart rate greater than 110 or less than 50 | Have you had any chest pain in the past 24 hours? If yes, please notify your primary care provider. If this is an emergency, please call 911. |
| Pulse-Ox reading less than 90% |  |
| No pulse ox reported for 1 consecutive days |  |
| No symptom survey answers for 2 consecutive days |  |

**Supplementary Table 2.** Comorbidities included as confounders in multivariable modeling. The following comorbidities were pre-specified by a direct acyclic graph to be relevant confounders, and included in doubly robust regression models and propensity score matching.

| **Condition** |
| --- |
| Atrial fibrillation |
| Hypertension |
| Coronary arterial disease or peripheral arterial disease |
| Diabetes |
| Obesity |
| Chronic Kidney Disease |
| Cancer |

**Supplementary Table 3.** Patient characteristics by diagnosis subgroups.

|  | **CHF** | | | | **COPD** | | |
| --- | --- | --- | --- | --- | --- | --- | --- |
|  | **Control**, N = 145 | **RPM**, N = 106 | **p-value** | | **Control**, N = 76 | **RPM**, N = 34 | **p-value** |
| ***Baseline Demographics*** | | | | | | | |
| Age, median (IQR) | 78 (71, 84) | 76 (66, 84) | 0.04 |  | 74 (67, 80) | 68 (62, 74) | 0.04 |
| Female gender, n (%) | 66 (46%) | 61 (58%) | 0.06 |  | 48 (63%) | 25 (74%) | 0.30 |
| Race, White, n (%) | 109 (75%) | 74 (70%) | 0.30 |  | 64 (84%) | 25 (74%) | 0.20 |
| Race, Black, n (%) | 36 (25%) | 32 (30%) | 0.30 |  | 12 (16%) | 9 (26%) | 0.20 |
| Any Insecurity, n (%) | 16 (11%) | 13 (12%) | 0.80 |  | 10 (13%) | 5 (15%) | >0.90 |
| Smoking, ever, n (%) | 109 (75%) | 64 (60%) | 0.01 |  | 71 (93%) | 32 (94%) | >0.90 |
| Medicaid, n (%) | 3 (2.1%) | 18 (17%) | <0.001 |  | 6 (7.9%) | 13 (38%) | <0.001 |
| ***Baseline Health Care Utilization Year Prior to Admission, median (IQR)*** | | | | | | | |
| Number of Admissions | 2 (1, 3) | 2 (1, 3) | 0.80 |  | 2 (1, 3) | 2 (1, 4) | 0.40 |
| Number of Office Visits | 8 (5, 13) | 9 (6, 13) | 0.20 |  | 6 (4, 10) | 7 (5, 11) | 0.30 |
| Number of Specialist Visits | 4 (2, 8) | 4 (2, 7) | 0.60 |  | 2 (0, 3) | 2 (1, 5) | 0.20 |
| Number of ED Visits without Admission | 0 (0, 1) | 0 (0, 1) | 0.50 |  | 0 (0, 1) | 1 (0, 1) | 0.50 |
| ***Baseline Medical Diagnoses, n (%)*** | | | | | | | |
| Systolic Heart Failure | 77 (53%) | 54 (51%) | 0.70 |  | 4 (5.3%) | 6 (18%) | 0.07 |
| Diastolic Heart Failure | 99 (68%) | 73 (69%) | >0.90 |  | 30 (39%) | 12 (35%) | 0.70 |
| COPD | 50 (34%) | 46 (43%) | 0.20 |  |  |  |  |
| Atrial Fibrillation | 85 (59%) | 49 (46%) | 0.05 |  | 20 (26%) | 8 (24%) | 0.80 |
| Hypertension | 142 (98%) | 97 (92%) | 0.02 |  | 60 (79%) | 29 (85%) | 0.40 |
| Coronary or Peripheral Arterial Disease | 114 (79%) | 60 (57%) | <0.001 |  | 36 (47%) | 12 (35%) | 0.20 |
| Diabetes | 66 (46%) | 57 (54%) | 0.20 |  | 23 (30%) | 16 (47%) | 0.09 |
| Obesity | 73 (50%) | 55 (52%) | 0.80 |  | 25 (33%) | 15 (44%) | 0.30 |
| Chronic Kidney Disease | 115 (79%) | 81 (76%) | 0.60 |  | 31 (41%) | 13 (38%) | 0.80 |
| Cancers, excluding non-metastatic skin cancers | 38 (26%) | 24 (23%) | 0.50 |  | 17 (22%) | 8 (24%) | 0.90 |
| ***Medications at Time of Cohort Entry, median (IQR)*** | | | | | | | |
| Total Number of Prescriptions | 14 (11, 18) | 15 (11, 19) | 0.20 |  | 15 (12, 19) | 17 (13, 22) | 0.20 |
| Total GDMT Meds | 2 (1, 2) | 2 (1, 3) | 0.40 |  | 1 (0, 1) | 1 (1, 2) | 0.04 |
| Prescribed any inhaler | 0 (0, 1) | 0 (0, 1) | 0.90 |  | 2 (2, 3) | 2 (2, 3) | 0.40 |
| Prescribed Insulin | 0 (0, 0) | 0 (0, 1) | 0.20 |  | 0 (0, 0) | 0 (0, 0) | 0.50 |

**Supplementary Table 4**. Unadjusted outcomes by diagnosis subgroup.

|  | **CHF** | | | **COPD** | | |
| --- | --- | --- | --- | --- | --- | --- |
|  | **Control**, N = 145 | **RPM**, N = 106 | **FDR p-value** | **Control**, N = 76 | **RPM**, N = 34 | **FDR p-value** |
| Composite Outcome | 96 (66%) | 58 (55%) | 0.20 | 49 (64%) | 24 (71%) | 0.63 |
| Admission or Death | 84 (58%) | 49 (46%) | 0.20 | 40 (53%) | 19 (56%) | 0.80 |
| Death | 25 (17%) | 7 (6.6%) | 0.05 | 12 (16%) | 2 (5.9%) | 0.60 |
| ED Visit | 39 (27%) | 27 (25%) | 0.86 | 20 (26%) | 14 (41%) | 0.56 |
| Admission | 78 (54%) | 49 (46%) | 0.47 | 35 (46%) | 18 (53%) | 0.64 |
| Total Length of Stay for Admissions | 6 (3, 14) | 8 (4, 14) | 0.64 | 6 (4, 12) | 8 (3, 19) | 0.80 |
| Num. Office Visits in 1 Month | 1 (1, 2) | 1 (0, 3) | 0.90 | 1 (0, 2) | 1 (1, 2) | 0.60 |
| Num. Office Visits in 6 Month | 5 (3, 7) | 6 (4, 9) | 0.05 | 4 (2, 6) | 4 (2, 7) | 0.60 |
| Num. Office Visits with Specialist in 6 months | 5 (3, 7) | 6 (4, 9) | 0.05 | 4 (2, 6) | 4 (2, 7) | 0.60 |
| Time to Composite Outcome | 38 (17, 82) | 33 (9, 75) | 0.62 | 35 (15, 85) | 27 (13, 66) | 0.64 |
| Time to Admission or Death | 41 (16, 76) | 39 (9, 77) | 0.64 | 34 (13, 84) | 49 (15, 96) | 0.80 |
| Time to Death | 83 (41, 124) | 91 (76, 101) | 0.86 | 72 (54, 102) | 17 (12, 21) | 0.28 |
| Time to ED Visit | 70 (29, 105) | 48 (24, 105) | 0.62 | 86 (57, 133) | 34 (16, 70) | 0.28 |
| Time to Admission | 41 (19, 84) | 39 (9, 77) | 0.62 | 27 (11, 86) | 55 (16, 99) | 0.64 |

**Supplementary Table 5.** Adjusted outcomes across subgroups.

| ***Doubly Robust Regression Model*** | **OR** | **99% Low CI** | **99% High CI** | **FDR p-value** |
| --- | --- | --- | --- | --- |
| ***Population Enrolled for CHF RPM*** | | |  |  |
| Composite Outcome | 0.56 | 0.13 | 0.98 | 0.20 |
| Admission or Death | 0.64 | 0.16 | 1.13 | 0.22 |
| Death | 0.40 | 0.00 | 0.94 | 0.20 |
| ED Visit | 0.79 | 0.11 | 1.46 | 0.47 |
| Admission | 0.23 | 0.19 | 1.36 | 0.47 |
| ***Population Enrolled for COPD RPM*** | | |  |  |
| Composite Outcome | 1.09 | 0.00 | 2.89 | 0.89 |
| Admission or Death | 0.90 | 0.00 | 2.02 | 0.89 |
| Death | 0.41 | 0.00 | 1.18 | 0.88 |
| ED Visit | 1.62 | 0.00 | 3.79 | 0.88 |
| Admission | 1.09 | 0.00 | 2.55 | 0.89 |
|  |  |  |  |  |
| ***Time-to-Event Analysis*** | **HR** | **99% Low CI** | **99% High CI** | **FDR p-value** |
| ***Population Enrolled for CHF RPM*** | | | |  |
| Time to Composite Outcome | 1.05 | 0.66 | 1.68 | 0.79 |
| Time to Admission or Death | 1.09 | 0.65 | 1.83 | 0.79 |
| Time to Death | 1.14 | 0.13 | 9.90 | 0.79 |
| Time to ED Visit | 1.28 | 0.62 | 2.65 | 0.79 |
| Time to Admission | 1.14 | 0.67 | 1.94 | 0.79 |
| ***Population Enrolled for COPD RPM*** | | | |  |
| Time to Composite Outcome | 1.12 | 0.51 | 2.47 | 0.94 |
| Time to Admission or Death | 0.98 | 0.46 | 2.06 | 0.94 |
| Time to Death | N/A | N/A | N/A |  |
| Time to ED Visit | 2.08 | 0.55 | 7.84 | 0.64 |
| Time to Admission | 0.93 | 0.42 | 2.07 | 0.94 |

**Supplementary Table 6.** Sensitivity analyses for missing social determinants of health data. In our primary adjusted analysis, we set missing data to absent for insecurities related to housing, food, or living expenses. Our sensitivity analysis includes (1) setting all missing data to present, and (2) setting missing data to absent or present contingent on the outcome.

|  | **OR** | **99% Low CI** | **99% High CI** | **FDR p-value** |
| --- | --- | --- | --- | --- |
| ***Base case, missing data set to absent*** | | |  |  |
| Composite Outcome | 0.68 | 0.25 | 1.11 | 0.30 |
| Admission or Death | 0.76 | 0.29 | 1.24 | 0.43 |
| Death | 0.41 | 0.00 | 0.86 | 0.20 |
| ED Visit | 1.01 | 0.30 | 1.73 | 0.96 |
| Admission | 0.91 | 0.35 | 1.47 | 0.86 |
| ***Scenario 1, missing data set to present*** | | |  |  |
| Composite Outcome | 0.62 | 0.22 | 1.01 | 0.13 |
| Admission or Death | 0.71 | 0.26 | 1.15 | 0.25 |
| Death | 0.42 | 0.00 | 0.88 | 0.13 |
| ED Visit | 0.98 | 0.29 | 1.66 | 0.93 |
| Admission | 0.83 | 0.31 | 1.35 | 0.55 |
| ***Scenario 2, missing data set contingent on outcome*** | | | |  |
| Composite Outcome | 0.82 | 0.24 | 1.39 | 0.85 |
| Admission or Death | 0.88 | 0.31 | 1.46 | 0.85 |
| Death | 0.48 | 0.00 | 1.02 | 0.45 |
| ED Visit | 1.12 | 0.32 | 1.92 | 0.85 |
| Admission | 1.05 | 0.38 | 1.71 | 0.85 |

**Supplementary Table 7**. Adherence data based on outcome.

|  | **No Outcome**, N = 58*^1^* | **Outcome**, N = 82*^1^* | **p-value** |
| --- | --- | --- | --- |
| BP Adherence | 94% (78%, 98%) | 74% (44%, 93%) | <0.001 |
| Weight Adherence | 94% (82%, 98%) | 75% (29%, 94%) | <0.001 |
| Pulse Ox Adherence | 94% (77%, 98%) | 74% (50%, 93%) | <0.001 |
| Survey Adherence | 87% (60%, 95%) | 59% (20%, 87%) | <0.001 |
| Time Enrolled in Study, days | 56 (39, 76) | 52 (31, 87) | 0.70 |

**Supplementary Table 8.** Exploratory as-treated analysis for adjusted outcomes. Engagement was measured as an average of adherence to survey and individual vitals (i.e. weight, pulse ox, blood pressure). Analyses were performed with a doubly robust analysis with engagement as a dichotomous variable requiring an average adherence >90%, and a logistic regression with engagement as a continuous variable.

| ***Doubly robust model*** | **OR** | **99% Low CI** | **99% High CI** | **FDR p-value** |
| --- | --- | --- | --- | --- |
| Composite Outcome | 0.34 | 0.04 | 0.65 | 0.003 |
| Admission or Death | 0.26 | 0.01 | 0.51 | 0.002 |
| Death | 0.32 | <0.01 | 0.91 | 0.14 |
| ED Visit | 1.04 | <0.01 | 2.08 | 0.91 |
| Admission | 0.30 | 0.02 | 0.58 | 0.002 |

| ***Logistic regression model*** | **OR** | **99% Low CI** | **99% High CI** | **FDR p-value** |
| --- | --- | --- | --- | --- |
| Composite Outcome | 0.36 | 0.02 | 7.00 | 0.40 |
| Admission or Death | 0.22 | 0.02 | 3.55 | 0.27 |
| Death | 0.01 | <0.01 | 0.51 | 0.03 |
| ED Visit | 0.08 | <0.01 | 1.58 | 0.08 |
| Admission | 0.41 | 0.03 | 6.16 | 0.40 |
